# Operationalizing machine-assisted translation in healthcare

**DOI:** 10.1038/s41746-025-01944-0

**Published:** 2025-09-30

**Authors:** Ivan Lopez, David E. Velasquez, Jonathan H. Chen, Jorge A. Rodriguez

**Affiliations:** 1 Stanford University School of Medicine, Stanford, CA USA; 2 Stanford Department of Biomedical Data Science, Stanford, CA USA; 3 Division of General Internal Medicine, Brigham and Women’s Hospital, Boston, MA USA; 4 Division of Hospital Medicine, Stanford University School of Medicine, Stanford, CA USA; 5 Clinical Excellence Research Center, Stanford School of Medicine, Stanford, CA USA; 6 Department of Medicine, Stanford, CA USA; 7 Harvard Medical School, Boston, MA USA

**Keywords:** Machine learning, Health policy

## Abstract

Over 25 million U.S. patients with a non-English language preference face unsafe care because discharge instructions and other materials are rarely translated in time. Advances in translation assisted by large language models can close this gap, but implementation guidance is scarce. Using the Consolidated Framework for Implementation Research, we outline key considerations—innovation, individuals, inner setting, implementation process, and outer setting—to offer healthcare leaders and policymakers a practical roadmap for language model machine-assisted translation integration.

## Introduction

Patients with a non-English language preference (NELP) experience significant barriers to high-quality health care, resulting in higher emergency department visits and hospital readmission rates compared to patients with an English language preference^1,2^. These disparities are driven by multilevel factors, including insufficient use of interpretation services (real‑time spoken language support)^3–5^ as well as limited availability of bilingual providers^6–9^. Another key contributing factor is the lack of translated medical information (written text). When patients require translated medical information, including discharge instructions or informed consent forms, certified translators either craft the translated documents de novo or use computer-aided translation (CAT) software, such as translation memory tools, grammar checkers, or terminology managers. These processes are costly, labor-intensive, and lack necessary customization^10–12^. In instances where in-house expertise is insufficient, health systems rely on third-party companies for translations. Despite these workflows, health systems continue to fail to routinely translate vital documents for patients with NELP^12–14^, even though the Civil Rights Act of 1964 and Department of Health and Human Services (HHS) regulations require U.S. hospitals to provide reasonable access to language assistance services, including written translations^15^.

Machine-assisted translation (MAT) using large language models (LLMs) has the potential to bridge this gap. Before 2023, neural machine translation (NMT) was the leading automated translation technology^16^. However, NMT systems struggle with specialized terminology and generally offer limited contextual understanding, particularly on longer passages^17^. Meanwhile, existing CAT software is hampered by limited adaptability and static glossaries, requiring considerable effort from translators to refine each piece of text. In contrast, LLMs provide deeper contextual understanding, enabling them to process entire passages and handle vocabulary and linguistic nuances more precisely^18^. They also support multi-task learning, allowing them to translate, summarize, or simplify text—capabilities crucial for producing accessible patient materials^19^. Although a single-center study reported that LLM translation achieved results comparable to human translators for languages with abundant online resources, such as Spanish and Portuguese^20^, concerns remain regarding its accuracy in languages with less digital content. Examples include Quechua and Yorùbá, which are considered digitally underrepresented languages in machine translation research due to the scarcity of comprehensive online corpora^21,22^. Additionally, there remains a lack of guidance on how to safely and effectively implement LLM-supported MAT technology into healthcare, particularly as generative artificial intelligence (AI) tools in medicine expand^19,23–27^.

To advance care for the millions of individuals with NELP, health systems and policymakers must take the lead in implementing LLM-supported MAT. Section 1557 of the Affordable Care Act has already established a fundamental requirement: machine-generated translations must undergo human review before reaching patients^28^. Beyond this high‑level mandate, there is no detailed federal or professional‑body guidance on how to operationalize LLM‑based MAT (e.g., evaluation, privacy safeguards, or workflow integration), leaving health systems to devise their own policies. In this perspective, we apply the Consolidated Framework for Implementation Research (CFIR)^29^—a comprehensive implementation science framework encompassing five domains and up to 67 constructs—as a conceptual lens to examine these challenges and considerations. By mapping these factors across CFIR domains, we present a roadmap that can help health systems judiciously operationalize MAT (Tables 1–5).

**Table 1:** Innovation domain: examines characteristics of an intervention that influence its adoption, implementation, and sustainability

Areas that Impact the Implementation Gap	Barriers to Implementation	Potential Solutions
A. Patient privacy	Risk of exposing PHI to third-party vendors or unprotected APIs	Partner with model providers to obtain ZDR endpoints or run a private instance of an open-sourced model (e.g., LLaMA-3.3 8B-Instruct)
	Risk of fine-tuning a model and having translation drafts include erroneous PHI from other cases	Scrub fine-tuning datasets of PHI using tools (e.g., Stanford de-identifier)
B. LLM limitations	Need for awareness of LLM limitations like hallucinations, context loss, and bias that lead to inaccurate translations	Design comprehensive training modules for translation teams on LLM limitations and mitigation strategies
	Reliance on automated outputs, reducing human accountability	Implement features prompting users to justify non-edits, similar to EHR clinical decision support
	Digitally underrepresented languages are more likely to perform poorer in LLM-based MAT	Promote equity and language inclusivity by thoroughly testing all languages used in the health system’s MAT workflow
C. Operating costs	High financial burden of running open-source LLMs on-premises (e.g., GPU costs)	Run quantized versions (e.g., 8-bit or 4-bit) of LLMs to reduce GPU costs
	Resource constrained hospitals in smaller or rural settings struggle to obtain the finances for GPUs or the overhead costs of maintaining a compute cluster	Multiple hospitals or a regional health network can partner to share costs to build and maintain a HIPAA-compliant cluster (data sent to this cluster should first be de-identified using highly accessible methods, such as the Stanford de-identifier). Costs can then be allocated proportionally based on usage, ensuring that heavy users contribute a correspondingly larger share of the operating expenses

*PHI* protected health information, *MAT* machine-assisted translation, *ZDR* zero-data-retention, *LLM* large language model, *EHR* electronic health record, *GPU* graphics processing unit.

**Table 2:** Individuals domain: focuses on the characteristics of individuals involved in implementation, including their knowledge, beliefs, and personal attributes

Areas that impact the implementation gap	Barriers to implementation	Potential solutions
D. Translators	LLM-based MAT integration that disrupts established workflows	EHR/IT teams can partner with translation leads to add “auto-draft” capabilities directly into translators’ current workflows. This should include integrating existing translator tools into the MAT workflow, such as CAT software
		Design comprehensive training focusing on best practices when interacting with MAT tools, how to handle MAT outages, etc.
E. Clinicians	Poorly structured or jargon-laden clinical notes cause translation errors and reduce MAT accuracy	Encourage clinicians to improve the quality of written discharge summaries
		Enable a two-prompt approach where the MAT LLM uses a “preparation” prompt to clean up messy notes before applying the “translation” prompt
F. Patients	Mistrust or misunderstanding of AI-driven translations	Ensure transparency regarding the use of AI in translating patient documents
		Inform patients (via consent forms or discharge packets) that MAT is used, emphasizing final human verification
	Lack of patient engagement in refining MAT processes	Patient advisory boards can be leveraged to collect direct patient feedback on clarity and acceptability of translations
		Tailor MAT deployment from patient feedback by offering MAT primarily in areas where patients feel comfortable (e.g., adult vs. pediatric settings), expanding usage as trust grows

*MAT* machine-assisted translation, *LLM* large language model, *EHR* electronic health record.

**Table 3:** Inner Setting Domain: encompasses the organizational context in which implementation occurs, including structural characteristics, available resources, and culture

Areas that impact the implementation gap	Barriers to implementation	Potential solutions
G. Translator workforce	Current translator workloads are saturating teams and leading to lengthy turnaround times	LLM-based MAT should offer a completed draft for the translator to review as soon as the translation request is made in the EMR
	Risk that low-quality LLM outputs demand more effort to manually correct compared to de novo synthesis	LLM-based MAT should follow selective deployment beginning with note types and languages that have been shown to perform well in prospective pilot studies. Deployment could be expanded gradually as data is collected and fine-tuning on MAT models is performed
	Reliance on third-party vendors that do not use MAT in their workflows reduces possible gains in translation efficiency	Third-party vendors and health systems should negotiate contracts for vendors to refine MAT drafts rather than translate from scratch, preserving speed and leveraging external expertise
H. Organizational culture	Translators have concerns about job displacement or diminishing professional expertise	Reinforce a translator-in-the-loop model that emphasizes LLM outputs should be a first draft with the final review remaining the translator’s responsibility
		Maintain Section 1557 of the Affordable Care Act that says machine-generated translations must undergo human review before reaching patients
	Translators feel like they are losing their autonomy	Organizations should set clear MAT expectations with translation teams on a case-by-case basis. For instance, translators may opt to use MAT tools for some projects while foregoing them for others. Nevertheless, health systems should strive to increase MAT usage over time, especially when it demonstrably enhances efficiency and patient outcomes

*MAT* machine-assisted translation, *LLM* large language model.

**Table 4:** Implementation process domain: focuses on the active strategies and actions taken to adopt, execute, and sustain an intervention

Areas that impact the implementation gap	Barriers to implementation	Potential solutions
I. Integration	Lack of guidance on the effective integration of MAT	Aligning with Existing Workflows: Embed MAT features directly into current EHR or translation software to minimize disruptions for translators and clinicians
		Co-Designing with End Users: Collaborate with translators and clinicians to tailor prompts, interfaces, and error-check features based on real-world needs
		Retrospective Testing: Use secure offline data to identify errors and refine the model prior to live deployment
		Prospective Testing: Pilot small-scale rollouts for a single language or document type to gather feedback and measure error rates
		Plan for Deployment Infrastructure: Employ centralized data storage (e.g., BigQuery) with detailed logging to enable auditing, troubleshooting, and secure PHI handling
		Fine-Tuning to Meet Deployment Needs: Continuously update the LLM with new translator-approved data, targeting challenging note types or languages first
		Measuring Real-World Impact: Track turnaround time, adoption rates, and patient outcomes to gauge the model’s overall value and display these in data dashboards (e.g., Looker Studio)
J. Evaluation	Lack of guidance on the effective evaluation of MAT	Translation Quality: Apply frameworks like MQM periodically to assess accuracy, fluency, and style on a sample of translated documents
		Operational Metrics: Track turnaround times, the percentage of language-concordant materials, and error rates to inform iterative improvements
		Clinical Outcomes: Monitor readmission or mortality rates in specific patient cohorts to gauge the real-world effects of MAT on care quality
		Patient Understandability and Actionability: Use tools like PEMAT and patient advisory boards to ensure translations are clear, actionable, and culturally appropriate

*PHI* protected health information, *MAT* machine-assisted translation, *EHR* electronic health record, *LLM* large language model, *MQM* multidimensional quality metrics, *PEMAT* patient education materials assessment tool.

**Table 5:** Outer setting domain: examines external influences on implementation, including policies, incentives, and interorganizational networks

Areas that impact the implementation gap	Barriers to implementation	Potential solutions
K. Regulatory oversight	Need for regulatory oversight of MAT practices in healthcare	Regulatory bodies, like the Joint Commission, should extend their evaluations to examine whether MAT meets the same standards required for traditional translation methods
	There is no standard benchmark for comparing LLM-generated versus human-generated translations	Expand existing Section 1557 compliance checks to include accuracy benchmarks for LLM outputs that include evaluation metrics described in the “Implementation Process Domain”
L. Data	There is a lack of centralized, high-quality bilingual corpora for training and validation with emphasis on sufficient examples for digitally underrepresented languages	A consortium-based dataset could be built via state health agencies, academic medical centers, and smaller hospitals that pool resources to build a shared de-identified translation repository
M. National Policy on LLM use	Outdated national standards (e.g., CLAS) that do not address LLM-based translation needs	Update CLAS standards to include specific guidelines and mandates for LLM use in medical translation
	Need for amendments to section 1557 of the Affordable Care Act and its implications on MAT	Revise Section 1557 to incorporate detailed requirements for LLM use, ensuring guidelines on safeguarding patient data privacy, acceptable MAT error rates and types, baseline qualifications an LLM must meet before it can be used for MAT, and clear roles and conditions when MAT can be used independently and when human review is mandatory

*MAT* machine-assisted translation, *LLM* large language model, *CLAS* culturally and linguistically appropriate services.

## CFIR for machine-assisted translation

### I. Innovation domain

For machine-assisted translation to succeed, health systems must learn to manage LLM limitations. For example, LLMs tend to “hallucinate” (generate plausible but factually incorrect text)^30^, lose context (difficulty retrieving and using information located in the middle of long passages)^31^, and degrade human accountability (where users become reliant on the LLM and fail to evaluate its outputs)^32^. The implementation of LLM MAT must therefore include appropriate safeguards. Translators should be trained to recognize the common pitfalls (e.g., hallucinations, context loss, bias) of LLMs in MAT, and, to counteract human overreliance, translators could be intermittently prompted to justify their choice to leave a sentence unedited in a translation, similar to safety checks in electronic health records (EHRs) for potential medication-medication interactions^33^.

Patient privacy is another design quality that must be considered. Closed-source LLMs should never be used in ways that expose protected health information (PHI) via unprotected application programming interfaces. We recommend leveraging zero-data-retention (ZDR) endpoints^34^ (API interfaces that process each request in memory only and do not log or persist any input or output data) or private instances through industry partnerships. For example, Stanford Health Care has established a dedicated pathway to the Azure OpenAI Service under full institutional control, enabling high-throughput, privacy-compliant LLM queries^35^. Such a model supports an environment for LLM-based MAT while following data protection standards.

Cost considerations also play a significant role in deciding whether to adopt LLMs. Operating large open-source models at scale can be prohibitively expensive for resource-constrained health systems. For example, running a single NVIDIA A100 80GB GPU on a cloud platform (e.g., Google Cloud) may cost around $4.74 per hour (as of May 2025)^36^. Since payors don’t reimburse LLM use, these costs must be covered by an institution’s operating budget. Two factors, however, can offset that burden. First, as secure ZDR endpoints become more widely available, organizations can adopt a pay-as-you-go model that reduces both infrastructure demands and overall expenses. These options can help democratize access to powerful LLMs for health systems of varying sizes while maintaining patient privacy. Second, patients with NELP have consistently longer stays, higher readmission rates, and greater care costs when language-concordant services are lacking^37,38^. Investing in LLM-based MAT can reduce those downstream expenses and improve value-based care metrics that payors already incentivize, such as Centers for Medicare & Medicaid Services (CMS) readmission penalties.

### II. Individuals domain

For translators, health systems should accommodate existing workflows. Though workflows vary, a common one involves a staff member submitting a request, then a translator is assigned the document and works in the EHR—either from scratch or using templates. In some organizations, a second translator reviews the draft before the original translator finalizes formatting and delivers the material to the patient. LLM-based MAT can be woven into this existing workflow by integrating into the EHR, enabling automatic draft generation as soon as a translation request is initiated. The translator can open the draft within the same interface they already use to make edits. Compatibility with CAT tools, allowing translators to run grammar checks or insert pre-translated templates from a memory database directly into the EHR, ensures they retain access to their preferred resources, and if a second review is standard, that reviewer can still validate the final text.

On the clinician side, translation accuracy depends on the quality of the English text. High-quality notes are well-structured, free from spelling errors, and written in plain language. To enhance accuracy, clinicians should prepare high-quality discharge summaries. Alternatively, MAT workflow may use an LLM’s zero-shot capabilities^39^ to first refine messy or jargon-heavy notes via a “preparation” prompt, then generate a translation with a second prompt. This two-prompt approach can be automatically applied to all incoming English text or toggled on/off by the translation team. By improving the quality of the source text, health systems can reduce the risk of inaccuracies.

Patients are also key stakeholders in MAT implementation. To secure their buy‑in, health systems must be transparent about their use of MAT and proactively seek patient input. Strategies may include brief post‑visit surveys, patient advisory boards, and focus groups dedicated to specific languages to elicit language‑specific guidance. Involving patients in this way is essential for both routine quality‑improvement loops and for the formal evaluation of LLM‑based MAT (see “Implementation Process Domain”). Additionally, surveying patients about their comfort with LLM-supported translations in various care settings (e.g., adult versus pediatric) can help create a more patient-centered approach to MAT deployment.

### III. Inner setting domain

An important Inner Setting factor is the translator workforce, which in many health systems is too small to meet the increasing demand for timely translations. Translators often face excessive workloads, causing lengthy turnaround times and leaving some patients without materials in their preferred language. Integrating LLM-based MAT can offload the initial draft, shortening the translation cycle and easing the translator’s workload. This approach also allows organizations to reallocate translator time to more complex tasks. However, it is crucial to recognize that a poorly generated LLM translation may demand more effort to correct than a fully manual translation. A practical approach to mitigate this risk is to first identify which documents and language pairs MAT consistently handles well (e.g., Spanish routine discharge summaries) and where it struggles (e.g., Korean surgery informed consent forms). In the early stages, organizations might limit MAT to documents and languages where the model performs reliably while relying on human translations for more complex tasks. As data from these difficult cases accumulate, the model can be fine-tuned and iteratively improved, gradually expanding its applicability. Accuracy remains paramount: tracking turnaround times alongside quality metrics ensures faster translations still meet patient needs. By weaving these measures into routine workflows, translation teams can maintain both efficiency and quality. We detail fine-tuning and evaluation in the Implementation Process Domain section.

Many health systems rely on third-party translation services rather than (or in addition to) in-house translators. In-house teams may offer greater control over workflows, direct communication with clinicians, and familiarity with local patient populations, but they require sustained funding, staffing, and expertise across multiple languages. Third-party services can support smaller organizations who may lack specialized linguistic support, though they may introduce logistical hurdles such as limited oversight and slower turnaround times. Some of these challenges are more difficult to resolve, but others, like slower turnaround times, could be mitigated if third-party vendors adopt translation editing services. Rather than translating documents entirely de novo, an in-house LLM-based system could generate a first draft for the vendor to refine. This approach preserves the time-saving benefits of LLM-assisted translation while still leveraging the expertise and capacity of external resources.

Organizational culture is another key pillar of the Inner Setting. Health system leaders must emphasize that AI complements—rather than replaces—human translators, preserving a translator-in-the-loop model that aligns with Section 1557 of the Affordable Care Act^28^. This approach values the expertise of translators, many deeply rooted in local communities, and ensures patients with NELP receive the same high standard of care as English-speaking counterparts. By positioning human translators as indispensable for quality control, cultural adaptation, and patient engagement, health systems maintain equity while harnessing AI’s efficiency and scalability^40^.

### IV. Implementation process domain

This section outlines practical steps for integrating LLM-supported MAT, drawing on the implementation of the Crisis Message Detector-1 (CMD-1), an AI-driven tool for triaging mental health messages^41^, as an example of embedding AI solutions into existing clinical workflows.

1. Aligning with existing workflows: Introduce MAT within platforms or software that translators and clinicians already use, thereby preserving established workflows. For instance, embedding an MAT draft feature into the EHR can streamline adoption. In the CMD-1 project, researchers integrated their AI model into Slack, an application providers already relied on, to minimize workflow disruptions.
2. Co-designing with end users: Actively involve translators and clinicians in MAT design—refining prompts, interfaces, and workflows—to address bottlenecks and foster trust. For example, if translators want an interactive interface with grading buttons, feedback fields, or error flags, co-designing these features into the EHR can improve implementation success. In CMD-1, for example, clinicians set the risk tolerance for false positives and false negatives.
3. Retrospective testing: A critical first step in implementation is to evaluate MAT on retrospective data before deployment. In CMD-1, the team first tested the model on historical data in an offline environment. This safe environment enabled the identification of errors without compromising patient safety. For MAT, retrospective testing should follow LLM changes (e.g., fine-tuning) or significant updates to translator workflows.
4. Prospective testing: After retrospective evaluation, health systems could start with small-scale deployments—limited to one frequently requested language, document type, or a few translators—and track usability, error rates, and impact over a defined period (e.g., two weeks). Once implementation is proven effective under these conditions, they could expand coverage to additional languages, documents, or translators. Key takeaways from these pilots identify areas needing improvement and highlight where MAT struggles most (e.g., certain languages or note types), guiding selective deployment and future fine-tuning. Prospective testing should follow significant model updates (e.g., post-fine-tuning) or major workflow changes.
5. Plan for deployment infrastructure: Even the best-performing model can fail without robust technical and operational support. Health systems must first select a secure data-storage solution, such as BigQuery^42^, Snowflake^43^, or Azure^44^, to store PHI. Just as CMD-1 relied on a dedicated platform to store and audit crisis-detection outputs, MAT requires a similarly comprehensive logging framework. All types of translations should be logged, whether generated entirely by a human translator or initially drafted through the LLM-supported workflow and then refined by a human. Each translation request should log a unique identifier, timestamp, LLM used, the model’s initial translation, the final version, note type, target language, translator ID, flags for harmful outputs, and metrics such as generation and editing times. Storing all of this in a single row for each translation record enables auditing, failure analysis, and model fine-tuning as part of ongoing clinical quality improvement.
6. Fine-tuning to meet deployment needs: Health systems can use the comprehensive logging framework to fine-tune LLM-based translation models in a supervised manner^45^. Specifically, final translator-approved versions (paired with the original English texts) provide gold-standard data for train/validation/test splits. The model is trained on the “train” split and monitored on the “validation” split using automatic metrics such as chrF++ or COMET, then evaluated on the “test” split to avoid overfitting. If a health system is using an open-source MAT model, it can reduce computational overhead by adopting Parameter Efficient Fine-Tuning libraries, such as Low-Rank Adaptation, which updates only a minimal subset of the model’s weights^45^. By analyzing prospective testing results, health systems can identify note types or languages where the model struggles and target those for fine-tuning. Over time, this iterative process broadens the LLM’s capabilities, improving performance across diverse document types and languages. It is also important to note that fine-tuning efforts may evolve from solely optimizing for translation accuracy to incorporating translator preferences. Once the model consistently drafts high-quality translations, further fine-tuning—using approaches like Direct Preference Optimization^46^—can tailor outputs to better align with the needs of individual translators or entire teams.
7. Measuring real-world impact: Success should be defined by practical outcomes, such as faster translation turnaround times, higher user satisfaction (both for translators and patients), and improved patient outcomes, rather than technical metrics alone. Tracking adoption rates and downstream effects (e.g., patient comprehension or readmission rates) offers a more comprehensive view of the model’s value. These operational metrics can be monitored in real time using data dashboards such as Looker Studio^47^ to ensure the system meets clinical and patient needs.

Evaluation is a critical component of implementation that can be approached through multiple methods.

**Fig. 1:**
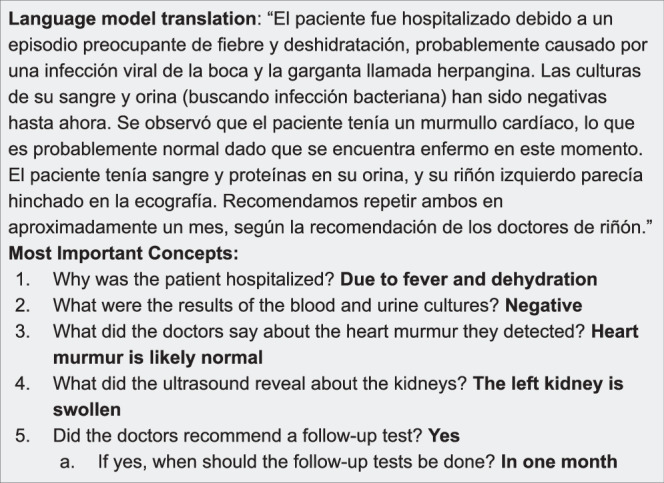
An illustrative patient-level evaluation workflow for LLM-supported MAT. Here, a Spanish discharge summary is followed by five open-ended questions covering key clinical concepts that will be answered by the patient. Each question has a “gold-standard” answer generated from the source English note, allowing evaluators to compare the patient’s responses with the intended information. This approach measures both the understandability and actionability of the translation by assessing how well patients grasp critical details from the LLM-generated text.

1. Translation quality: The Multidimensional Quality Metrics (MQM) framework—which rates accuracy, fluency, terminology, style, and locale appropriateness and tags each error as critical, major, or minor—provides a robust gauge of quality^48^. Other healthcare MT studies have used the validated 5-point fluency-adequacy-meaning-severity rubric which scores meaning preservation and grammar while labeling errors by clinical severity^49^. However, these frameworks are time-intensive and are best applied periodically (e.g., monthly) on a representative sample of translations. One way to form this sample is by computing sentence embeddings of the source text and selecting a diverse subset based on similarity scores^50^. Ideally, manual evaluation should be performed by the translation team, leveraging their expertise for nuanced feedback that guides ongoing model improvements. For routine monitoring, we recommend a combination of automated metrics: chrF++ for character‑level similarity^51^ and COMET for semantic adequacy and fluency^52^. BLEU, which measures n-gram overlap between the LLM output and the final human-approved text^53^, could be included for historical comparability. However, its correlation with human judgements is inconsistent, so it should be interpreted with caution and supplemented by newer metrics that show stronger alignment with human ratings^54^.
2. Operational metrics: Translation turnaround time and the proportion of patients with NELP who receive language-concordant discharge instructions should be used to measure system efficiency, pinpoint areas for workflow refinement, and ensure equitable care delivery across diverse language groups.
3. Clinical outcomes: Health systems need to track process and clinical outcomes. They can focus on measures prioritized by the CMS, such as readmission and mortality rates for conditions like heart failure or Chronic Obstructive Pulmonary Disease^1^. These outcomes can be tracked by extracting data from clinical notes^55,56^ or ICD-10 codes^57,58^ for patients with NELP.
4. Patient understandability and actionability: The Patient Education Materials Assessment Tool (PEMAT), which assesses the clarity, ease of understanding, and actionability of health care materials, could be used to evaluate MAT^59^. Similar to MQM, it is time-consuming to administer and best applied periodically (e.g., monthly) on a representative subset of translations^50^. PEMAT evaluation should be carried out by patients or patient advisory boards. Another approach to evaluation involves having a clinician identify five key points from a discharge summary, then convert those into open-ended questions. A patient representative reads the translated text and answers these questions (Fig. 1). By grading the accuracy of responses, teams gauge how well LLM-supported translations convey essential clinical information.

### V. Outer setting domain

As LLM adoption increases, organizations like the Joint Commission are needed to conduct independent quality assurance checks of MAT workflows. As part of its accreditation process, the Joint Commission assesses whether hospitals comply with Section 1557 of the Affordable Care Act, which mandates that patients with NELP receive appropriate language access services^28^. In doing so, the Joint Commission verifies that hospitals have processes in place to deliver patient materials that are accurate, timely, and linguistically appropriate. As health systems begin to integrate MAT, these evaluations can be extended to examine whether MAT meets the same standards required for traditional translation methods using evaluation methods described in the “Implementation Process Domain.”

Health systems and public health agencies should collaborate to create a shared clinical‑translation corpus, similar to open resources such as MIMIC‑IV, which provides de‑identified clinical notes for public research use^60^. Each participating institution (academic medical centers, community hospitals, rural and safety‑net facilities) would contribute de‑identified source notes across the full spectrum of document types (e.g., post‑operative instructions, discharge summaries, consent forms) paired with translator‑verified versions. The corpus would (i) maintain distinct training and held‑out test datasets and (ii) grow through a community‑driven process in which sites donate a small batch of new notes, de‑identified with methods such as “hiding in plain sight”^61^. Efforts should be made to collect translations for digitally underrepresented languages. Over time, this iterative, collaborative approach will enrich clinical translation data available for fine‑tuning and will maintain an evolving benchmark for evaluating LLM-based MAT.

New policy guidelines are needed to support MAT implementation, beginning with national standards for LLM use. The HHS Office of Minority Health developed the National Standards for Culturally and Linguistically Appropriate Services (CLAS) in Health and Health Care. These standards, last updated in 2013, provide a framework for culturally and linguistically appropriate care^62^ but require revision to ensure accurate, reliable, and timely MAT. HHS should also revisit Section 1557 of the Affordable Care Act to aid implementation. It's May 6, 2024 rule revision was the first to address machine translation^28^, but further guidance is needed on safeguarding patient data, acceptable error rates, and critical error reporting. Given the substantial variation in LLM performance across different languages, policy guidelines need to be language-specific, rather than a single blanket standard. For instance, health systems could rank languages based on an LLM’s internal representations^63^, examine the distribution of languages in the LLM’s training corpus (when made publicly available)^64^, or evaluate performance on an in-house testing dataset to determine the languages in which a given model meets MAT standards. The next rule revision must set baseline qualifications for LLMs used in MAT, ensuring that only suitable models are implemented.

## Conclusion

Equitable access to linguistically concordant information remains a major unmet need for the millions of people in the United States who prefer a language other than English. Machine translation with LLMs offers promising solutions—reducing translator workload, shortening turnaround times, and extending translation services to resource-constrained settings. However, it also introduces new challenges, including context loss and inaccuracies in digitally underrepresented languages. For safe, equitable outcomes, health systems must continuously evaluate accuracy, address biases, and refine workflows. Moving forward, hybrid effectiveness-implementation studies across various clinical settings^65^ will be essential for determining whether LLM-based MAT truly reduces language-related disparities in practice.

## Data Availability

No datasets were generated or analysed during the current study.
